# Supplementary figures and images for: Slug Mediates MRP2 Expression in Non-Small Cell Lung Cancer Cells
Source: Biomolecules. 2022 Jun 9;12(6):806. doi: 10.3390/biom12060806 (PMC9220960; doi:10.3390/biom12060806)

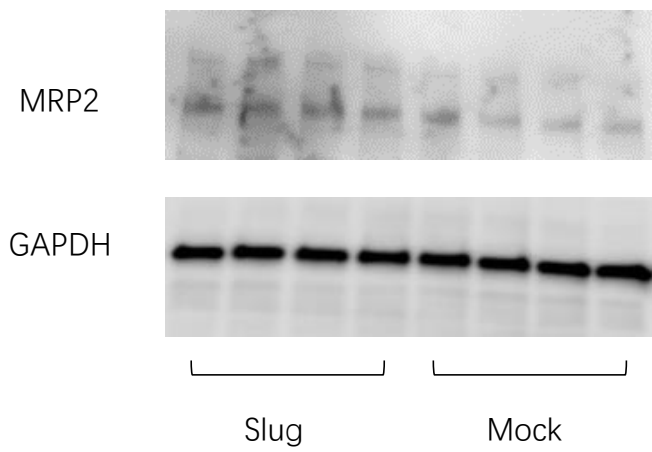

Supplement: Supplementary file 1 [file biomolecules-12-00806-s001.zip › biomolecules-1729379-supplementary.pdf]
